# Dementia-related risk associated with endocrine and targeted therapy strategies in breast cancer: a real-world pharmacovigilance analysis

**DOI:** 10.3389/fphar.2026.1855112

**Published:** 2026-10-23

**Authors:** Ruolin Cui, Yulian Yin, Hongfeng Chen

**Affiliations:** 1 Longhua Clinical Medical College, Shanghai University of Traditional Chinese Medicine, Shanghai, China; 2 Department of Traditional Chinese Medicine, Xinhua Hospital, Shanghai Jiao Tong University School of Medicine, Shanghai, China; 3 Department of Traditional Chinese Medicine Breast Disease, Longhua Hospital Affiliated to Shanghai University of Traditional Chinese Medicine, Shanghai, China

**Keywords:** breast cancer, dementia, endocrine therapy, pharmacovigilance, targeted therapy

## Abstract

**Background:**

In clinical practice, adjuvant endocrine and targeted (ET) therapy strategies for hormone receptor-positive (HR+) breast cancer are constantly evolving. The long-term cognitive impact of such adjuvant treatment on patients remains unclear.

**Methods:**

This study mined data from the FDA Adverse Event Reporting System (FAERS) from 2010Q1 to 2025Q2 to examine the relationship between ET therapy strategies and adverse events (AEs) related to dementia. Standardized *Medical Dictionary for Regulatory Activities* (MedDRA) Queries (SMQs) containing “broad” and “narrow” preferred terms (PTs) for dementia were used to systematically identify AE reports associated with dementia. The disproportionality analysis was conducted using the Empirical Bayes Geometric Mean (EBGM) calculated by the Multi-Item Gamma Poisson Shrinker (MGPS) and the proportional reporting ratio together with chi-square (PRR, χ2).

**Results:**

As a whole, risk signals are negative in narrow or broad dementia (narrow: EBGM05 1.27, PRR 1.32 [χ^2^ 5.75]; broad: EBGM05 1.02, PRR 1.03 [χ^2^ 2.88]). In subgroup analyses, positive signals of narrow dementia were identified for anastrozole monotherapy (n = 20, EBGM05 4.00, PRR 4.68 [χ^2^ 53.20]) and exemestane monotherapy (n = 9, EBGM05 2.10, PRR 2.64 [χ^2^ 8.85]) in female adult breast cancer cases, as well as for anastrozole monotherapy in reporters over 50 years old (n = 20, EBGM05 2.52, PRR 3.67 [χ^2^ 39.87]).

**Conclusion:**

This FAERS-based pharmacovigilance analysis identifies subgroup-specific narrow-spectrum dementia safety signals for aromatase inhibitor monotherapy in breast cancer patients, most notably anastrozole in women aged over 50 years. These hypothesis-generating observations warrant prospective validation.

## 1 Introduction

Breast cancer remains the most prevalent malignancy among women globally. In 2020, an estimated 2.26 million new cases were reported worldwide, accounting for approximately 24.5% of all new female cancer cases (Sung et al., 2021). Approximately two-thirds of breast cancer patients are hormone receptor-positive (HR+), and systemic treatment for this subgroup is anchored by endocrine and targeted (ET) therapy strategies (Burstein et al., 2018).

In early-stage HR+ breast cancer, adjuvant endocrine therapy reduces the 10-year recurrence risk by 30%–50% and improves overall survival by 15%–20% (Early Breast Cancer Trialists' Collaborative Group, 2015). For metastatic HR+ breast cancer, endocrine therapy agents are often combined with targeted drugs to extend progression-free survival and preserve quality of life (Cristofanilli et al., 2016). In clinical practice, six major classes of agents play key roles in HR+ breast cancer adjuvant treatment, divided into endocrine and novel targeted therapies (Korde et al., 2021). Endocrine therapies suppress tumor growth by modulating hormone signals (Zelnak and O’Regan, 2015), including aromatase inhibitors (AI) that block estrogen synthesis, selective estrogen receptor modulators (SERM) that regulate estrogen receptor activity, selective estrogen receptor downregulators (SERDs) that degrade estrogen receptors, and GnRHas that inhibit gonadotropin secretion (Ascione et al., 2024). Novel targeted therapies focus on specific tumor pathways and consist of cyclin-dependent kinase 4/6 inhibitor (CDK4/6i) that blocks cell cycle progression (Braal et al., 2020) and mammalian target of rapamycin inhibitors (mTORis) that suppress mTOR-mediated tumor proliferation (Browne et al., 2024). These agents are administered as monotherapies or in combination, with treatment strategies dependent on disease stage, hormonal status, and therapeutic goals (Wardell et al., 2015), which typically range 5–10 years (Goldhirsch et al., 2013).

ET therapy strategies have transformed the management of HR+ breast cancer; however, AEs associated with long-term use have increasingly emerged (Hall et al., 2021), including osteoporosis (Gomes and Vale, 2025), insomnia (Henry et al., 2024), and cognitive decline (Bakoyiannis et al., 2015)*.* Regarding the impact of breast cancer adjuvant ET therapy on cognitive functions, previous studies have focused more on traditional endocrine therapy drugs such as AI and SERM, suggesting that AI may cause a decline in memory and concentration by inhibiting the synthesis of estrogen (Bender et al., 2007), while tamoxifen may have a protective effect due to its partial estrogenic activity (Blanchette et al., 2020). Moreover, the cognitive impacts vary among patients of different age groups undergoing long-term AI treatment. For novel targeted agents, the risk of dementia-related AEs remains undefined. Even less is known about the dementia-related safety of combined ET therapy strategies, which are widely used in clinical practice.

Pharmacovigilance studies have assessed the impact of endocrine therapy on the risk of dementia in breast cancer patients (Yuste et al., 2025; 2026). However, pharmacovigilance studies on dementia risks of endocrine combined with targeted therapy for breast cancer remain limited.

Therefore, considering the potential clinical value, in-depth investigation is required to clarify the correlation between medications used in breast cancer ET therapy and AEs related to dementia. Such analysis will help deliver a complete profile of dementia-associated AEs linked to ET therapy in adult female patients with breast cancer and offer evidence for clinical decisions.

## 2 Materials and methods

### 2.1 Data source

We conducted a pharmacovigilance study on dementia-related events associated with drugs of breast cancer ET therapy based on the FAERS database. Pharmacovigilance databases of FAERS, a publicly available database of safety reports submitted by patients, healthcare professionals, and pharmaceutical companies (Zhou et al., 2023), enable the large-scale real-world detection of drug–AE associations for identifying cognitive risks of breast cancer endocrine therapy, thus solving limitations of small sample sizes and short follow-up in clinical trials. Since the FAERS database is accessible to the public and patient records are anonymized and de-identified, ethical clearance and informed consent are not required for this study. AEs reported in the FAERS database were coded based on PT codes from MedDRA 28.0, with different PTs grouped into SMQs. SMQs further improve analysis reliability by classifying dementia-related adverse events into “narrow” and “broad” categories to reduce coding-related misclassification bias (Mozzicato, 2007).

### 2.2 Data processing procedure

All AE records submitted to the FAERS database from 1 January 2010 to 30 June 2025 were retrieved and processed. Duplicate entries were removed before further analysis; the full screening procedure is illustrated in Figure 1. This study only included female patients aged 18 years or older with a confirmed diagnosis of breast cancer. Drugs for breast cancer ET therapy, including SERMs (tamoxifen and toremifene), AIs (anastrozole, letrozole, and exemestane), GnRHas (goserelin and leuprorelin), SERDs (fulvestrant and elacestrant), CDK4/6i (abemaciclib), and mTORi (everolimus), were used as keywords of primary suspected drugs to obtain further cases. The remaining records underwent additional screening to exclude cognitive impairment events potentially attributed to confounding factors, in accordance with the following criteria. First, reports were excluded if they contained PTs associated with cognitive dysfunction, including brain metastases, meningeal metastases, encephalitis, and meningitis. Second, cases were omitted if the reported treatment indications included brain metastases, meningeal metastases, central nervous system (CNS) disease progression, or dementia secondary to other systemic disorders. Thirdly, records were excluded if patients received concomitant medications known to induce cognitive impairment, including benzodiazepines (e.g., alprazolam), anticholinergics (e.g., oxybutynin), and chemotherapeutic agents (e.g., cisplatin). Although these non-endocrine confounder therapies could be controlled to some extent, completely removing their effects is challenging. After the above steps, each remaining case was evaluated for the occurrence of the target adverse event category, which was established using MedDRA SMQs based on narrow and broad PTs associated with dementia-related adverse events in MedDRA 28.0. The exact terminology applied is provided in the Appendix. Ultimately, the final dataset eligible for subsequent statistical analysis was established.

**FIGURE 1:**
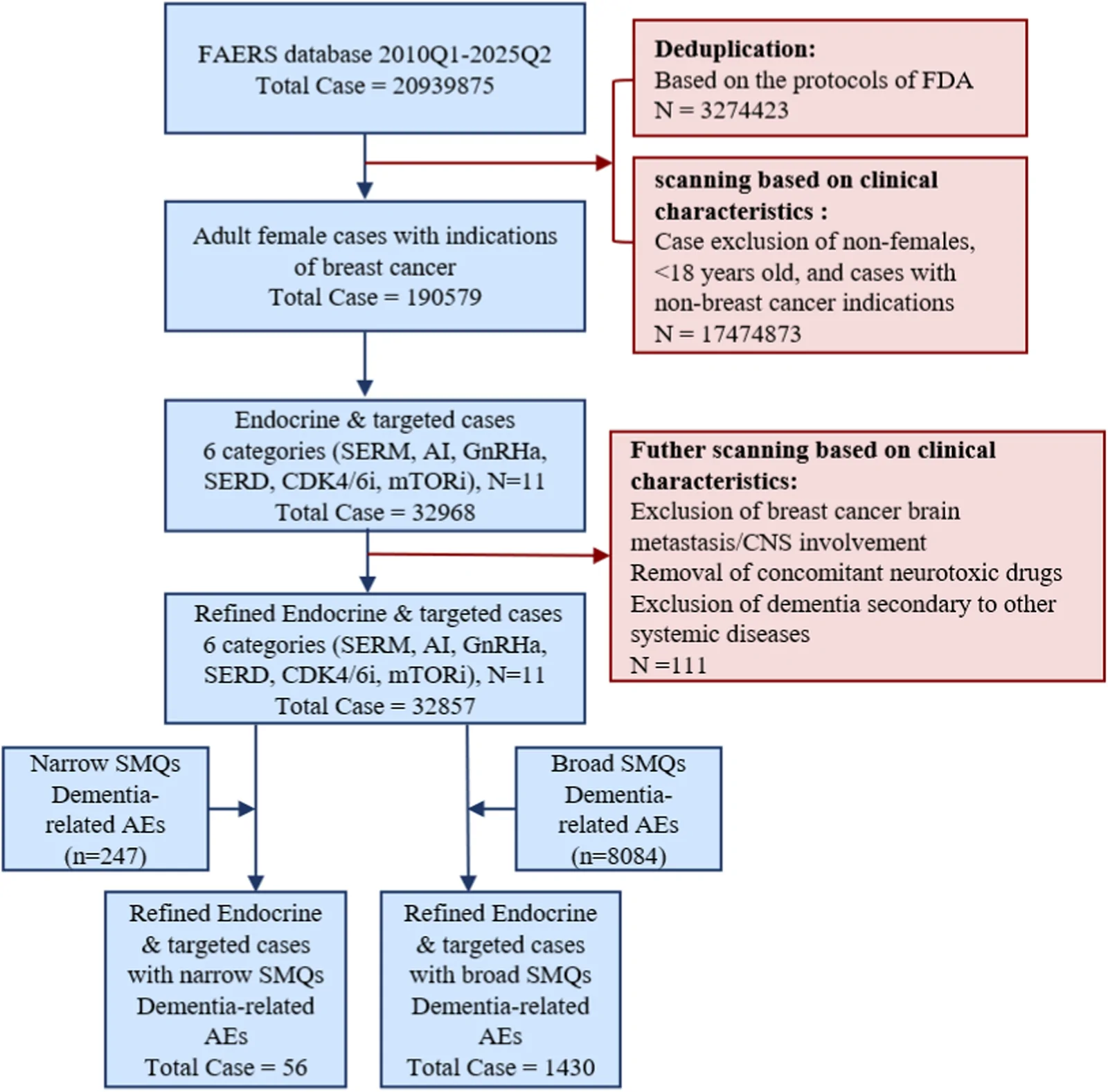
Flowchart of case selection for endocrine and targeted therapy‐related dementia in adult female breast cancer patients from FAERS (2010Q1–2025Q2).

### 2.3 Data analysis

A population-based pharmacovigilance study using a case/non-case approach was applied to analyze the risk of dementia for breast cancer ET therapy drugs. This method is widely applied in pharmacovigilance research for the detection of potential drug safety signals (Ahmad et al., 2022). Mathematically, the case/non-case design operates by comparing the incidence of the target AE in subjects exposed to a given study medication (defined as cases) against the frequency of the same AE in subjects without exposure to that agent (defined as non-cases) (Rothman et al., 2004; Sakaeda et al., 2013). This case/non-case framework is analogous to a case-control study, and its outcomes can be quantified using the Multi-Item Gamma Poisson Shrinker (MGPS) algorithm and proportional reporting ratio (PRR) along with the corresponding chi-square (χ2). In our study, disproportionality assessment was implemented by computing the Empirical Bayes Geometric Mean (EBGM) derived from MGPS and PRR (χ2). The EBGM quantifies the ratio of the observed number of AE reports for a given drug to the number expected under the null hypothesis of no association, calculated as EBGM = a (a + b + c + d)/(a + c)/(a + b), in which a is the number of reports of dementia-related AE for breast cancer ET drugs, b represents the reports for breast cancer ET drugs without reporting dementia, c is the number of the reports of dementia for all other drugs, and d represents the number of reports for all other drugs without reporting dementia. Unlike the raw proportional reporting ratio, the MGPS algorithm applies Bayesian shrinkage to the observed disproportionality estimate, which pulls extreme estimates toward the null for drugs with small sample sizes, thereby reducing false-positive signals. The lower 95% confidence bound of the EBGM (EBGM05) was used as the primary threshold for signal detection. To reduce false-positive signals, a positive disproportionality signal was conservatively defined as meeting both thresholds simultaneously (EBGM05 ≥ 2.0 and PRR>2 with χ^2^ > 4), consistent with the dual-criterion approach commonly used in FAERS pharmacovigilance studies (Sakaeda et al., 2013). Results that met only one of the two thresholds were considered exploratory signals, suggestive of a possible association requiring further confirmation. For the purposes of visualization, signal strength was further graded by EBGM05 magnitude as strong (EBGM05 ≥ 2.0), moderate (1.5≤EBGM05 < 2.0), and weak (1.2≤EBGM05 < 1.5) following established pharmacovigilance practice for representing disproportionality estimates on a continuous gradient (Wu et al., 2024).

Subgroup analyses were performed to explore dementia-related AE signals under different clinical scenarios. First, stratification by treatment strategies was conducted, including monotherapy subgroups and combination therapy subgroups. Second, age-stratified analyses were performed across three predefined age bands: 18–50 years, 50–65 years, and ≥65 years. Third, for strategies with positive or exploratory signals in the initial disproportionality analyses, further age-specific analyses were conducted within their clinically applicable age ranges.

Time-to-onset analysis was performed using reports in which both “drug_start_time” and “event_time” were documented. The median time to onset and interquartile range (IQR) were calculated for both narrow and broad dementia-related AEs. The distribution of onset time intervals was also summarized to characterize the temporal pattern of dementia-related events following ET therapy initiation.

For CDK4/6 inhibitors, abemaciclib was selected as the primary agent for the main analysis due to its more consistent and complete AE reporting in the FAERS database, as well as its documented higher blood-brain barrier penetrance compared with other agents in this class (Li et al., 2021), making it the most pharmacologically relevant agent for investigating cognitive adverse events. To assess the robustness of CDK4/6 inhibitor-related findings, a sensitivity analysis was additionally performed including all three clinically available agents (abemaciclib, palbociclib, and ribociclib) as a combined class, using the same disproportionality analysis methodology.

A sensitivity analysis using a cognition-restricted broad SMQ was performed to assess the robustness of broad signals. This analysis incorporated all three clinically available agents (abemaciclib, palbociclib, and ribociclib) as a combined class to ensure comprehensiveness. We excluded 29 non-specific preferred terms (sleep, psychotic, and mood-related) from the original 60 PTs and retained 31 cognition-specific PTs (Appendix). Disproportionality metrics and signal thresholds matched the primary analysis.

### 2.4 Statistical analysis

The normality of the data was evaluated by Kolmogorov–Smirnov test. Data from a normal distribution were expressed as mean ± standard deviation, while data from non-normal distributions were expressed as median (interquartile range [IQR]). Categorical variables were expressed as their frequencies and percentages. Statistical analyses were performed using R (version 4.5.1).

## 3 Results

### 3.1 Data overview

A total of 20,939,875 cases were available on the FAERS database. Of these, 3,274,423 were excluded based on the exclusion criteria. We then filtered the query for female adults indicated for breast cancer and identified 190,579 cases in FAERS during the analytical period. Overall, 32,968 cases were found to be related to drugs of breast cancer ET therapy, among which 32,746 cases were retained for further analysis after excluding cases with other potential cognitive function-influenced factors. Some 247 cases related to narrow dementia-related AEs and 8,084 cases with broad dementia-related AEs were documented. Among them, drugs of breast cancer ET therapy were identified as the suspected drug causing narrow dementia-related AEs in 56 cases and broad dementia-related AEs in 1,430 cases.

### 3.2 General characteristics

The demographic characteristics of the breast cancer ET therapy-associated dementia cases are presented in Table 1. In terms of age group, those over 65 years old accounts for highest proportion in both cases with narrow and broad SMQs, but it is more prominent in cases with narrow SMQs, accounting for 83.9%. In cases of documented weight, the median weight for cases with narrow SMQs is lower than that of cases with broad SMQs. Regarding time to onset, the median time to onset of cases with narrow SMQs is 120.00 (IQR 44.75–388.00) days, is longer than that of cases with broad SMQs, which is 77.00 (IQR 16.00–282.00) days. In terms of outcome, non-fatal outcomes are more common in cases with narrow SMQs, while in cases with broad SMQs, more outcomes are unknown or missing. Regarding reporter sources, healthcare providers reported 58.93% of the narrow cases, while non-health-care providers reported 52.52% of the broad cases. In addition, most cases were reported from Great Britain (39.28%) in cases with narrow SMQs and from countries other than Great Britain and United States (47.06%) in cases with broad SMQs. The distribution of reports across years shows a highly statistically significant difference between cases with narrow and broad SMQs. Notable temporal variation was observed in narrow SMQ reports, with a disproportionate concentration of cases in 2013 (32.14%), likely reflecting year-to-year variability in reporting patterns rather than a true change in underlying risk. The proportions of PTs in breast cancer ET therapy cases with narrow and broad SMQs are shown in Figure 2.

**TABLE 1:** Clinical characteristics of cases with endocrine and targeted therapy-related dementia.

Characteristics	Narrow SMQs (n = 56)	Broad SMQs (n = 1,430)	*p*-value
Age group (years)			0.012*
18 ≤ and < 50	1 (1.80%)	177 (12.40%)	​
50 ≤ and < 65	8 (14.30%)	482 (33.70%)	​
≥65	47 (83.90%)	771 (53.90%)	​
Unknown or missing	0 (0%)	0 (0%)	​
Age			0.978
Mean (SD)	75.58 (10.02)	—	​
Median (IQR)	—	66.00 (57.00-73.00)	​
Weight group(kg)			0.771
<50	0 (0%)	49 (3.43%)	​
50 ≤ and < 100	23 (41.07%)	587 (41.05%)	​
≥100	0 (0%)	60 (4.20%)	​
Unknown or missing	33 (58.93%)	734 (51.33%)	​
Weight			0.01*
Median (IQR)	58.50 (53.00-71.700)	68.9 (58.40-80.10)	​
Onset time(days)			0.035*
Median (IQR)	120.00 (44.75-388.00)	77.00 (16.00-282.00)	​
Unknown or missing	38 (67.86%)	853 (59.7%)	​
Outcome			<0.001**
Fatal	4 (7.10%)	99 (6.92%)	​
Non-fatal	51 (91.10%)	1,021 (71.40%)	​
Unknown or missing	1 (1.80%)	310 (21.68%)	​
Reporter			<0.001**
Healthcare professional	33 (58.93%)	557 (38.96%)	​
Physician	22 (39.29%)	284 (19.86%)	​
Pharmacist	2 (3.57%)	71 (4.97%)	​
Other	9 (16.07%)	202 (14.13%)	​
Non-healthcare professional	19 (33.93%)	751 (52.52%)	​
Consumer	19 (33.93%)	748 (52.31%)	​
Lawyer	0 (0%)	3 (0.21%)	​
Unknown or missing	4 (7.14%)	122 (8.52%)	​
Reported country			<0.001**
US	17 (30.36%)	596 (41.68%)	​
GB	22 (39.28%)	161 (11.26%)	​
Other countries	17 (30.36%)	604 (47.06%)	​
Unknown or missing	0 (0%)	69 (4.83%)	​
Reported year			<0.001**
2010	2 (3.57%)	47 (3.29%)	​
2011	1 (1.79%)	54 (3.78%)	​
2012	1 (1.79%)	49 (3.43%)	​
2013	18 (32.14%)	93 (6.50%)	​
2014	0 (0%)	65 (4.55%)	​
2015	3 (5.36%)	81 (5.66%)	​
2016	4 (7.14%)	106 (7.41%)	​
2017	1 (1.79%)	71 (4.97%)	​
2018	3 (5.36%)	93 (6.50%)	​
2019	7 (12.50%)	112 (7.83%)	​
2020	0 (0%)	81 (5.66%)	​
2021	2 (3.57%)	113 (7.90%)	​
2022	7 (12.50%)	123 (8.60%)	​
2023	3 (5.36%)	147 (10.28%)	​
2024	4 (7.14%)	125 (8.74%)	​
2025	0 (0%)	70 (4.90%)	​
Unknown or missing	0 (0%)	0 (%)	​

*p < 0.05, **p < 0.001.

**FIGURE 2:**
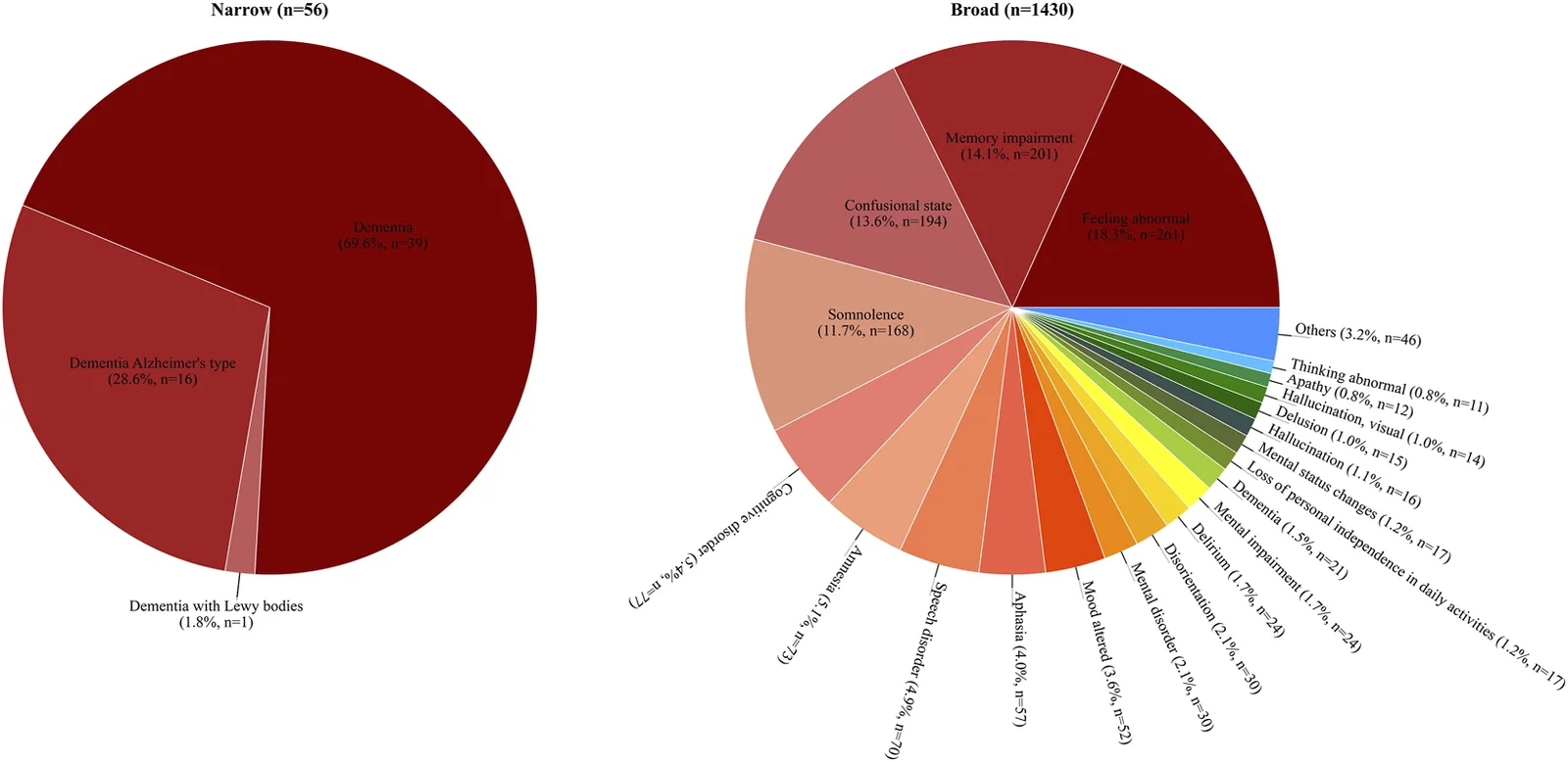
PTs of endocrine and targeted therapy‐related dementia in adult female breast cancer patients.

### 3.3 Disproportionality analysis

The EBGM05 was 1.27 and the PRR (χ2) was 1.32 (5.75) for narrow dementia-related AEs associated with drugs of breast cancer ET therapy, demonstrating that no overreporting of narrow dementia-related AEs was identified in drugs of breast cancer ET therapy within adult female breast cancer patients. Similarly, broad dementia-related complications were not over-reported in the adult female breast cancer patients administrated with drugs of breast cancer ET therapy (EBGM05, 1.02; PRR 1.03, χ2 2.88).

### 3.4 Subgroup analysis

Subgroup analyses by treatment strategy are presented as EBGM05 (Figure 3). Among monotherapy strategies, both anastrozole (n = 20, EBGM05 = 4.00, PRR = 4.68, χ^2^ = 53.20) and exemestane (n = 9, EBGM05 = 2.10, PRR = 2.64, χ^2^ = 8.85) met the pre-specified criteria for positive disproportionality signals of narrow dementia, with EBGM05 values above 2.0 and PRR values above 2.0 (all χ^2^ > 4). Among combination strategies, two exploratory signals were identified. AI plus CDK4/6i for narrow-dementia (n = 5, EBGM05 = 1.60, PRR = 2.41, χ^2^ = 4.06) and AI plus GnRHa combined with CDK4/6i for broad-dementia (n = 11, EBGM05 = 1.74, PRR = 2.01, χ^2^ = 5.84) both met the PRR criterion but not the EBGM05 ≥ 2.0 threshold.

**FIGURE 3:**
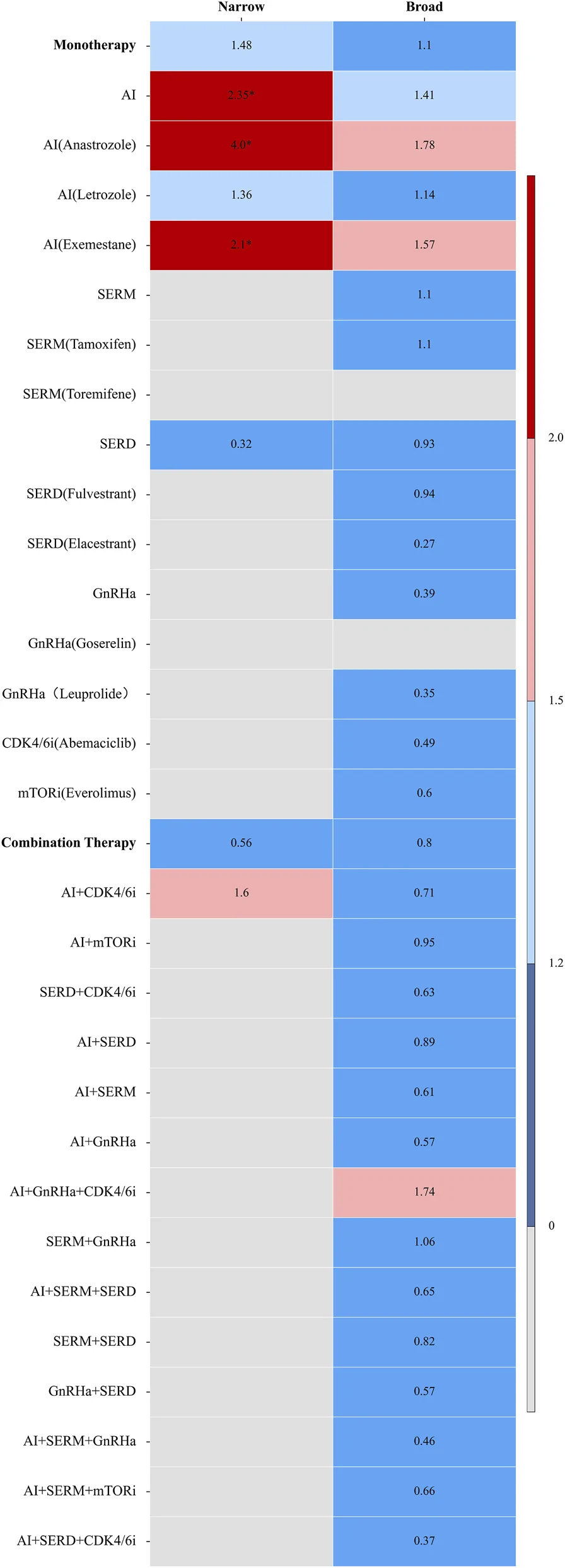
Subgroup disproportionality analysis by treatment strategy (EBGM05 heatmap). Signal strength is graded by EBGM05 magnitude: strong (EBGM05≥2.0, marked with*), moderate (1.5≤EBGM05<2.0), and weak (1.2≤EBGM05<1.5).

Among cases of all age groups, the PRRs for dementia (narrow and broad) were less than 2.0 in all specified age subgroups, of which the over-65-year-old group for narrow dementia (PRR 1.93) approached but did not reach the pre-specified PRR >2 threshold (Figure 4).

**FIGURE 4:**
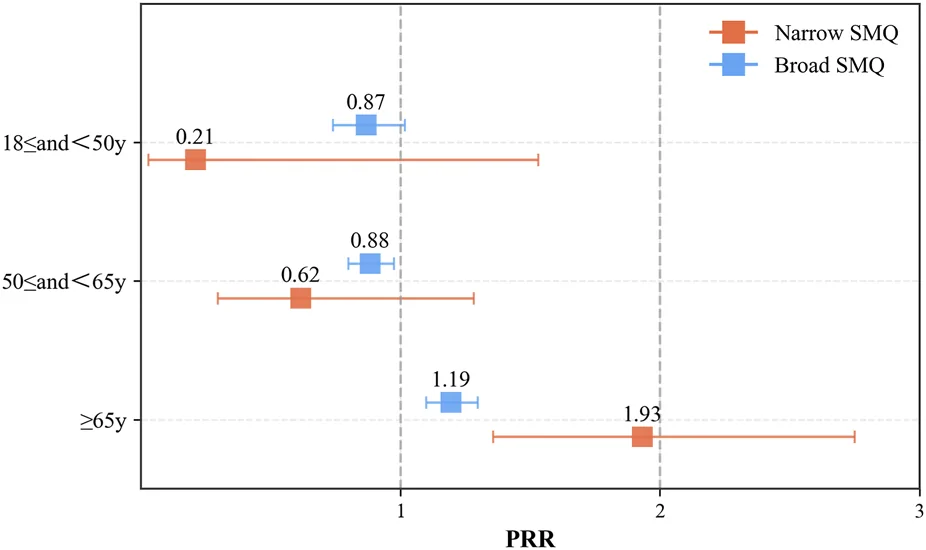
Subgroup disproportionality analysis of dementia following endocrine and targeted therapy compared to all other drugs within adult female breast cancer patients from FAERS by age (PRR). A positive PRR signal was defined as PRR>2 with χ^2^>4.

Further age-specific analyses for regimens with positive or exploratory signals were conducted within their clinically applicable age ranges. For anastrozole monotherapy in reporters aged over 50 years, the narrow-dementia signal remained positive with 20 reported cases (EBGM05 = 2.52, PRR = 3.67, χ^2^ = 39.87), meeting both pre-specified thresholds. For the combination of AI plus GnRHa with CDK4/6 inhibitors in patients aged 18–50 years, ten cases of broad-dementia were reported, yielding an exploratory signal that met the PRR criterion but not the EBGM05 ≥ 2.0 threshold (EBGM05 = 1.56, PRR = 2.64, χ^2^ = 10.50) (Figure 5). Full 2 × 2 contingency tables (a, b, c, and d values) for all positive and exploratory subgroup signals are provided in Supplementary Table S1.

**FIGURE 5:**
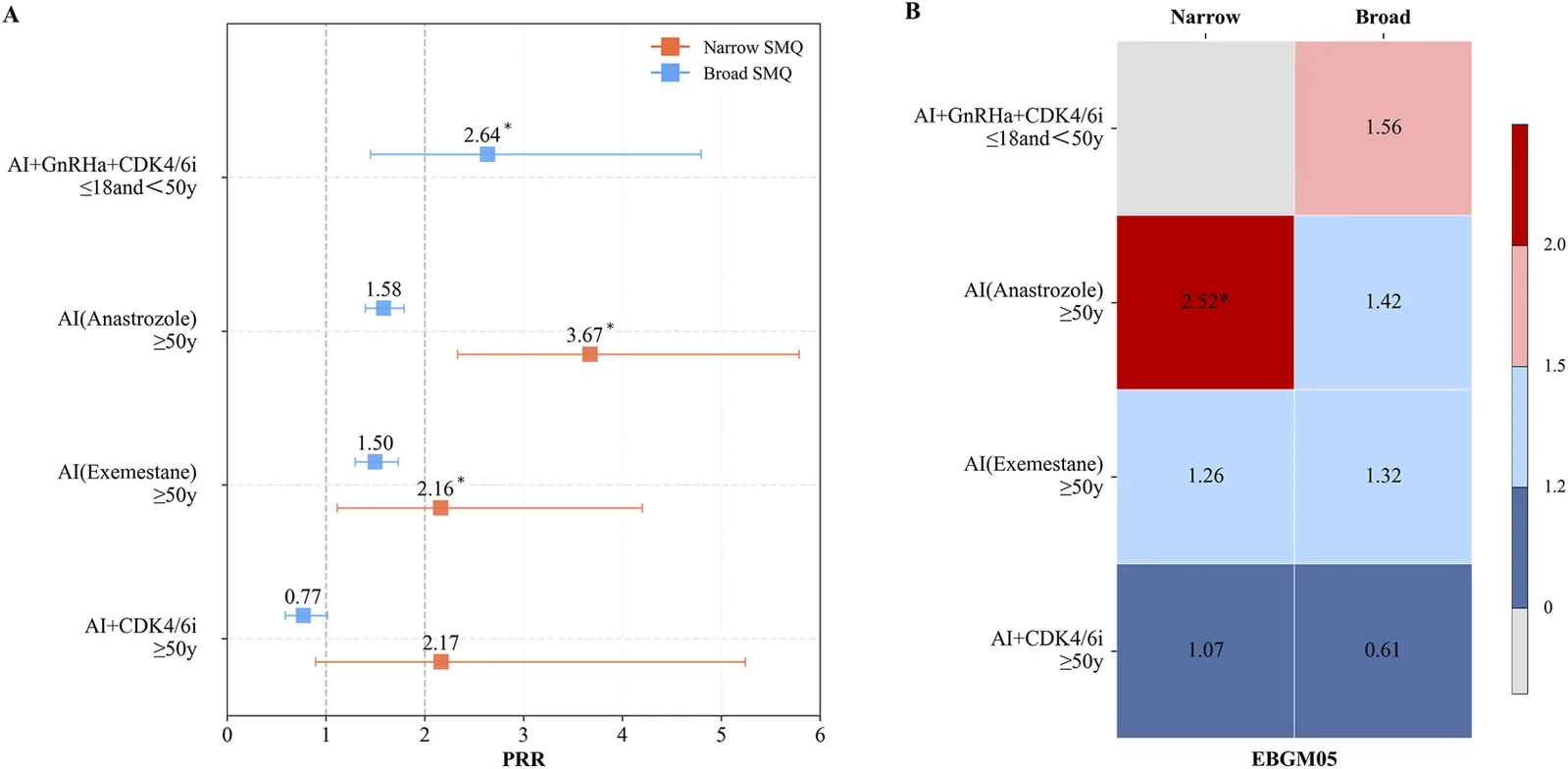
Subgroup disproportionality analysis of dementia following endocrine and targeted therapy compared to all other drugs within adult female breast cancer patients from FAERS by specific therapy strategy crossing age. **(A)** Forest plot showing PRR values with 95% confidence intervals, * indicates positive signals meeting PRR>2 with χ^2^>4. **(B)** Heatmap showing EBGM05 signal strength, Signal strength is graded by EBGM05 magnitude: strong (EBGM05≥2.0, marked with*), moderate (1.5≤EBGM05<2.0), and weak (1.2≤EBGM05<1.5).

### 3.5 Sensitivity analysis of CDK4/6 inhibitor class

All exploratory signals identified in the primary abemaciclib-only analysis demonstrated attenuation following inclusion of palbociclib and ribociclib. Specifically, the narrow-SMQ exploratory signal for the AI plus CDK4/6 inhibitor combination in adult female breast cancer reporters fell below both detection thresholds (EBGM05: 1.60 to 1.14; PRR: 2.41 to 1.61; χ^2^ = 9.29). The broad-SMQ exploratory signal for the AI plus GnRHa plus CDK4/6 inhibitor triple combination was similarly abolished in both the all-adult population (EBGM05: 1.74 to 0.26; PRR: 2.01 to 0.41; χ^2^ = 15.76) and the 18–50 years age subgroup (EBGM05: 1.56 to 0.59; PRR: 2.64 to 1.00; χ^2^ < 0.01). These findings demonstrate that the observed dementia-related disproportionality signals are agent-specific to abemaciclib rather than reflecting a class-wide effect of CDK4/6 inhibitors.

### 3.6 Sensitivity analysis of broad SMQ specificity

Restriction to cognition-specific terms resulted in a 42.6% reduction in total broad-dementia cases, from 8,084 to 4,640 across the overall study population. Notably, the exploratory broad-SMQ signal previously observed for the AI plus GnRHa plus CDK4/6 inhibitor combination was no longer present after SMQ refinement (n = 24, EBGM05 = 0.63, PRR = 0.95, χ^2^ = 0.03), falling well below both detection thresholds. One additional exploratory signal was identified for the quadruple combination of AI plus CDK4/6 inhibitor plus GnRHa plus SERM (n = 7, EBGM05 = 1.91, PRR = 4.52, χ^2^ = 14.18), which met the PRR threshold but not the EBGM05 criterion.

### 3.7 Time to onset

Generally, the median adverse event onset interval for dementia was 120.00 (IQR 44.75-388.00) days for reports with narrow SMQs, and 77.00 (IQR 16.00-282.00) days for broad cases (Table 1). The times to onset are summarized in Figure 6. In addition, most narrow dementia-related AEs occurred within 179 days of breast cancer ET therapy initiation, while most broad dementia-related AEs occurred within 539 days of ET therapy initiation.

**FIGURE 6:**
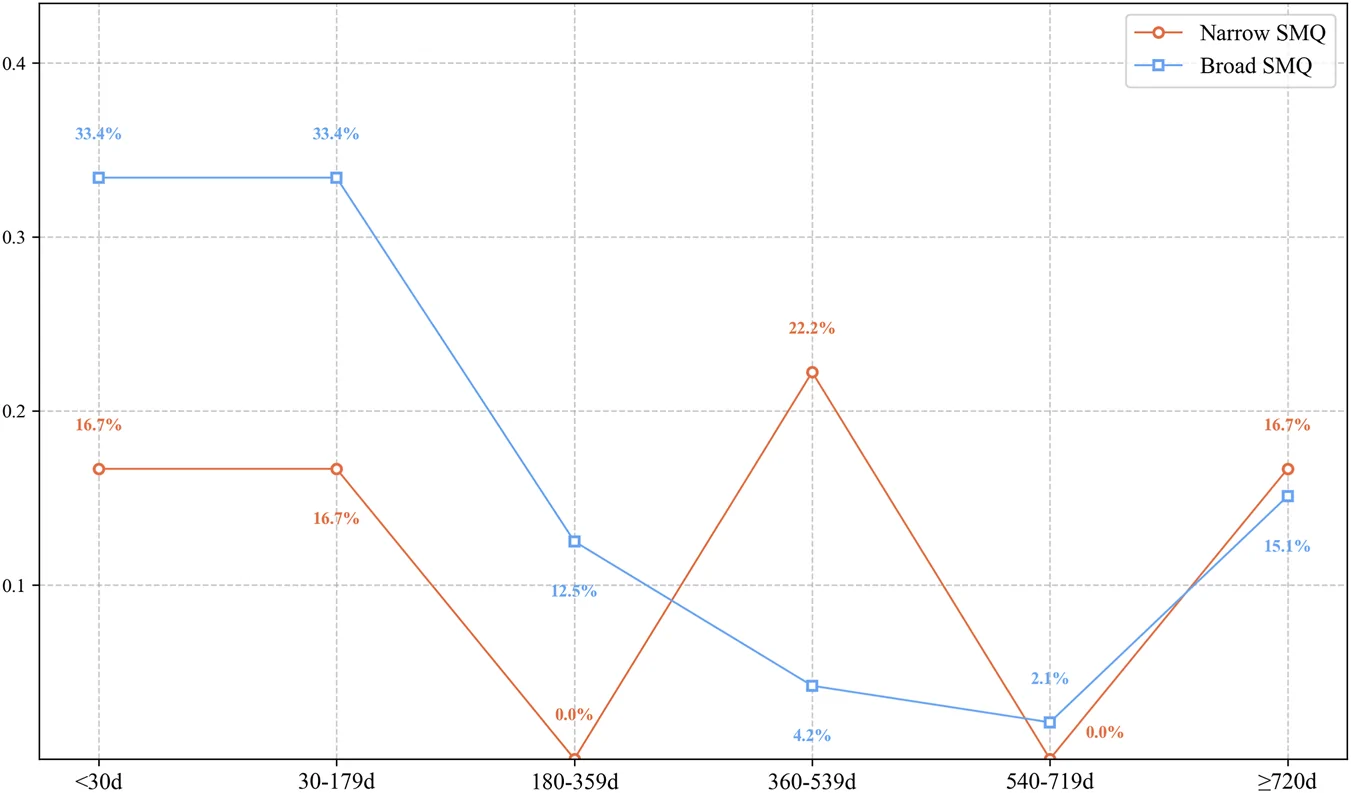
Time to onset of endocrine and targeted therapy‐related demented AEs.

## 4 Discussion

This study comprehensively assesses the dementia-related risks associated with long-term usage of endocrine and targeted therapy strategies among adult female breast cancer patients through the analysis of real-world data, providing evidence-based support for clinical medication management. Our findings of the association between anastrozole and dementia risk are consistent with existing evidence that AIs may induce cognitive changes. In a cross-sectional study, different cognitive effects were identified between patients using AIs and those using tamoxifen (Abdallah et al., 2022). Age affected treatment-related cognitive complaints, which is consistent with our age-stratified results (Abdallah et al., 2022). An umbrella review confirmed the association of endocrine therapy with cognitive impairment, particularly affecting memory and executive function, while highlighting inconsistent overall dementia risk due to study heterogeneity (Oliva et al., 2024); this explains our neutral overall signal.

This study also addresses evidence gaps in research on novel targeted therapies. A systematic review confirmed the elevated adverse event risk of CDK4/6 inhibitors but did not include dementia-specific data, highlighting the general scarcity of evidence concerning combination strategies (Guan et al., 2024). The preliminary risk signal we observed for combined AIs, GnRHas, and CDK4/6 inhibitor therapy in younger patients offers the first real-world indication of potential cognitive risks associated with such combinations.

The risk associated with anastrozole in patients over age 50 may mechanistically originate from estrogen depletion. AIs reduce extragonadal estrogen synthesis, and postmenopausal women already exhibit diminished estrogen levels that are critical for synaptic plasticity and neuroprotection (Li et al., 2015). In younger patients, AI-induced peripheral estrogen suppression, combined with GnRHa-mediated artificial menopause, may create a compounded hormonal deficit (Prévot et al., 2023). For CDK4/6 inhibitors, preclinical evidence of cellular senescence induction in neuroblastoma models (Rader et al., 2013), coupled with the documented blood–brain barrier penetrance of abemaciclib (Wander et al., 2022) and the established role of cyclin D in hippocampal cell proliferation, suggests a potential impact on neural homeostasis. The convergence of profound estrogen suppression with potential CDK4/6 inhibitor-related neural effects may exert synergistic effects on cognitive function, consistent with prior reviews of endocrine therapy and CDK4/6 inhibitor-related cognitive outcomes (Kjoe et al., 2022). These mechanistic insights provide a biological framework for interpreting the pharmacovigilance signals observed in our study, which remain hypothesis-generating and warrant further investigation (Yu et al., 2024).

The sensitivity analysis employing a refined, cognition-restricted broad SMQ provides important context for the interpretation of our findings. The 42.6% reduction in total broad-dementia cases following the exclusion of sleep-related, psychotic, and mood-related terms is consistent with the well-documented challenge of defining cognitive outcomes in pharmacovigilance databases, wherein neuropsychiatric symptoms are frequently multifactorial and may stem from disease burden, concomitant medications, or psychological distress rather than dementia itself (Ahles et al., 2012). The attenuation of broad-SMQ exploratory signals further substantiates the substantial contribution of non-specific terminology to broad SMQ case counts. Notably, the exploratory signal for the AI plus GnRHa plus CDK4/6 inhibitor combination strategy was completely abrogated following SMQ refinement—a finding concordant with the higher disease burden and polypharmacy characteristic of patients receiving combination therapy. A novel exploratory signal emerged for the quadruple AI plus CDK4/6 inhibitor plus GnRHa plus SERM combination after broad SMQ refinement; the small number of cases and the relative rarity of this treatment strategy in clinical practice indicate that this is a preliminary safety signal requiring further validation in larger datasets.

As these findings represent hypothesis-generating safety signals from pharmacovigilance data rather than confirmed causal associations, they may inform personalized clinical monitoring strategies. Consideration could be given to more frequent cognitive monitoring for patients over 50 years old receiving anastrozole, although clinical decisions should be individualized. For premenopausal women for whom ovarian suppression is clinically indicated, GnRHa-containing strategies remain a standard component of endocrine therapy; the potential cognitive risk signal observed in this study should therefore be integrated into individualized risk–benefit discussions, taking into account disease stage, recurrence risk, and patient preferences, rather than constituting a contraindication to GnRHa use. This approach aligns with endocrine therapy safety guidelines that emphasize the management of long-term side effects (Loibl et al., 2024). Furthermore, pharmacovigilance systems should implement age-stratified reporting of cognitive adverse events. This practice is recommended for accurately capturing therapy-related risks within heterogeneous patient populations.

In addition, this study found a high incidence of narrow SMQs within 179 days of endocrine and targeted therapy initiation. The time-to-onset pattern provides empirical support for differentiated monitoring strategies. For patients aged over 50 receiving anastrozole monotherapy, a focus on screening for cognitive functions is recommended using tools such as the Montreal Cognitive Assessment (MoCA) within 1–6 months of treatment initiation. For young patients undergoing a combination of AIs, GnRHas, and CDK4/6 inhibitors, the duration of cognitive function follow-up should be extended to 18 months, with early intervention to avoid long-term cognitive impairment.

Several limitations should be considered when interpreting our findings. First, as a FAERS-based pharmacovigilance study, our analysis is subject to inherent limitations of spontaneous reporting systems (Sakaeda et al., 2013), including underreporting of mild cognitive events, notoriety bias (the Weber effect, whereby newly approved drugs receive disproportionately higher reporting rates in the early post-marketing period), stimulated reporting, and variations in reporting patterns by source type and geographic region. This is particularly relevant for abemaciclib, which received FDA approval in 2017 and may therefore exhibit artificially elevated reporting rates during the later part of our study period. Second, the lack of denominator data precludes the calculation of absolute incidence rates, and the retrospective, spontaneous nature of the data prevents definitive causal inference. Third, although we excluded cases with known cognitive-impairing concomitant medications (e.g., benzodiazepines, anticholinergics, and chemotherapy) and comorbidities (e.g., brain metastases and CNS disease progression), residual confounding from unmeasured baseline characteristics, lifestyle factors, and additional comorbidities cannot be fully excluded (Oliva et al., 2024). Additionally, missing or incomplete clinical data (e.g., dosage, treatment duration, and disease stage) limit more nuanced analyses such as dose-response assessments, and potential residual duplicate reports may inflate case counts despite our de-duplication procedures. Finally, sensitivity analysis incorporating palbociclib and ribociclib demonstrated that the observed dementia signal was specific to abemaciclib-containing regimens and did not extend to the broader CDK4/6 inhibitor class. Differences in pharmacological properties, particularly blood–brain barrier penetrance, across individual agents should therefore be considered when interpreting these findings.

## 5 Conclusion

This FAERS-based pharmacovigilance analysis demonstrates the absence of an overall dementia safety signal for ET therapy strategies in breast cancer but reveals subgroup-specific narrow-dementia signals with AI monotherapy—most notably anastrozole in patients aged over 50. These real-world observations are hypothesis-generating and merit further prospective investigation to confirm their clinical relevance.

## Data Availability

Publicly available datasets were analyzed in this study. These data can be found at: https://fis.fda.gov/extensions/FPD-QDE-FAERS/FPD-QDE-FAERS.html.

## Appendix

### Dementia-like and related events

In this study, we systematically searched AE reports coded using the 28.0 version of the MedDRA using SMQs with “narrow” and “broad” preferred terms (PTs) related to dementia.

The narrow PTs used were as follows: “dementia,” “dementia Alzheimer’s type,” “dementia of the Alzheimer’s type, uncomplicated,” “dementia of the Alzheimer’s type, with delirium,” “dementia of the Alzheimer’s type, with delusions,” “dementia of the Alzheimer’s type, with depressed mood,” “dementia with Lewy bodies,” “frontotemporal dementia,” “mixed dementia,” “presenile dementia,” “vascular dementia.”

The broad PTs used were as follows: “abnormal behavior,” “abulia,” “affect lability,” “agnosia,” “amnesia,” “amnestic disorder,” “anterograde amnesia,” “apathy,” “aphasia,” “apraxia,” “behavior disorder,” “cerebral atrophy,” “change in sustained attention,” “clock drawing test abnormal,” “cognitive disorder,” “confusional state,” “delirium,” “delusion,” “delusion of theft,” “delusional disorder, jealous type,” “delusional disorder, unspecified type,” “disinhibition,” “disorientation,” “disturbance in social behavior,” “executive dysfunction,” “Fazekas scale abnormal,” “feeling abnormal,” “hallucination,” “hallucination, auditory,” “hallucination, visual,” “hallucinations, mixed,” “illusion,” “impaired reasoning,” “inappropriate affect,” “intelligence test abnormal,” “initial insomnia,” “judgement impaired,” “learning disorder,” “loss of personal independence in daily activities,” “memory impairment,” “mental disorder,” “mental impairment,” “mental status changes,” “mood altered,” “neuropsychological symptoms,” “neuropsychological test abnormal,” “personality change,” “prodromal Alzheimer’s disease,” “pseudohallucination,” “psychotic behavior,” “psychotic disorder,” “somnolence,” “speech disorder,” “suspiciousness,” “symbolic dysfunction,” “thinking abnormal,” “transient global amnesia,” “vascular cognitive impairment,” “visual cortex atrophy,” “visuospatial deficit.”

The refined cognition-restricted broad PTs used for sensitivity analysis were as follows: “agnosia,” “amnesia,” “amnestic disorder,” “anterograde amnesia,” “aphasia,” “apraxia,” “cerebral atrophy,” “change in sustained attention,” “clock drawing test abnormal,” “cognitive disorder,” “confusional state,” “delirium,” “disorientation,” “executive dysfunction,” “Fazekas scale abnormal,” “impaired reasoning,” “intelligence test abnormal,” “judgement impaired,” “loss of personal independence in daily activities,” “memory impairment,” “mental impairment,” “neuropsychological symptoms,” “neuropsychological test abnormal,” “prodromal Alzheimer’s disease,” “speech disorder,” “symbolic dysfunction,” “thinking abnormal,” “transient global amnesia,” “vascular cognitive impairment,” “visual cortex atrophy,” “visuospatial deficit.”
