# Supplementary material for: Dementia-related risk associated with endocrine and targeted therapy strategies in breast cancer: a real-world pharmacovigilance analysis
Source: Front Pharmacol. 2026 Oct 23;17:1855112. doi: 10.3389/fphar.2026.1855112 (PMC13739796; doi:10.3389/fphar.2026.1855112)
Supplement: Supplementary file 1 [file Supplementaryfile1.docx]

Supplementary Material

# 1 Supplementary Data

Supplementary Material should be uploaded separately on submission. Please include any supplementary data, figures and/or tables.

Supplementary material is not typeset so please ensure that all information is clearly presented, the appropriate caption is included in the file and not in the manuscript, and that the style conforms to the rest of the article.

# 2 Supplementary Figures and Tables.

## 2.1 Supplementary Figures

**Supplementary Figure 1.** Flowchart of case selection for endocrine and targeted therapy-related dementia in adult female breast cancer patients from FAERS (2010Q1–2025Q2).

**Supplementary Figure2.** PTs of endocrine and targeted therapy-related dementia in adult female breast cancer patients.

**Supplementary Figure3.** Subgroup disproportionality analysis by treatment strategy (EBGM05 heatmap). Signal strength is graded by EBGM05 magnitude: strong (EBGM05≥2.0, marked with*), moderate (1.5≤EBGM05<2.0), and weak (1.2≤EBGM05<1.5).

**Supplementary Figure4.**Subgroup disproportionality analysis of dementia following endocrine and targeted therapy compared to all other drugs within adult female breast cancer patients from FAERS by age (PRR). A positive PRR signal was defined as PRR>2 with χ²>4.

**Supplementary Figure5.**Subgroup disproportionality analysis of dementia following endocrine and targeted therapy compared to all other drugs within adult female breast cancer patients from FAERS by specific therapy strategy crossing age. (A) Forest plot showing PRR values with 95% confidence intervals, * indicates positive signals meeting PRR>2 with χ²>4. (B) Heatmap showing EBGM05 signal strength, Signal strength is graded by EBGM05 magnitude: strong (EBGM05≥2.0, marked with*), moderate (1.5≤EBGM05<2.0), and weak (1.2≤EBGM05<1.5).

**Supplementary Figure6.** Time to onset of endocrine and targeted therapy-related demented AEs.

**Supplementary Table S1.** Full 2×2 contingency tables for positive and exploratory dementia safety signals across subgroups.

| Drugs strategy | Age Group | SMQ | a | b | c | d | EBGM05 | PRR | χ² | Signal Level |
| --- | --- | --- | --- | --- | --- | --- | --- | --- | --- | --- |
| Anastrozole monotherapy | ≥18years | Narrow | 20 | 3,504 | 227 | 186,828 | 4.00 | 4.68 | 53.20 | Positive |
| Exemestane monotherapy | ≥18years | Narrow | 9 | 2,682 | 238 | 187,650 | 2.10 | 2.64 | 8.85 | Positive |
| AI class (all agents) | ≥18years | Narrow | 42 | 13,166 | 205 | 177,166 | 2.35 | 2.75 | 38.91 | Positive |
| Anastrozole monotherapy | >50 years | Narrow | 20 | 3,274 | 223 | 143,489 | 2.52 | 3.67 | 39.87 | Positive |
| AI + CDK4/6i | ≥18years | Narrow | 5 | 1,612 | 242 | 188,720 | 1.60 | 2.41 | 4.06 | Exploratory |
| AI + GnRHa + CDK4/6i | ≥18years | Broad | 11 | 118 | 8,073 | 182,377 | 1.74 | 2.01 | 5.84 | Exploratory |
| AI + GnRHa + CDK4/6i | 18–50 years | Broad | 10 | 121 | 1,251 | 42,191 | 1.56 | 2.64 | 10.50 | Exploratory |
| Exemestane monotherapy | >50 years | Narrow | 9 | 2,508 | 234 | 144,255 | 1.26 | 2.16 | 5.74 | Exploratory |

*Notes: a, exposed cases (target drug+dementia); b, exposed non-cases (target drug+no dementia); c, unexposed cases (no target drug+dementia); d, unexposed non-cases (no target drug+no dementia). Positive signal: defined as meeting both thresholds simultaneouslyEBGM05 ≥ 2.0 and PRR > 2 with χ² > 4. Exploratory signal: defined as meeting only one of the two thresholds (either EBGM05 ≥ 2.0 or PRR > 2 with χ² > 4), suggestive of a possible association that requires further confirmation. SMQ, Standardized MedDRA Query; EBGM05, lower 95% confidence limit of the Empirical Bayes Geometric Mean; PRR, Proportional Reporting Ratio; AI, Aromatase Inhibitor; GnRHa, Gonadotropin-Releasing Hormone agonist; CDK4/6i, Cyclin-Dependent Kinase 4/6 inhibitor.*
